# Persistent Afebrile Abdominal Pain: An Unusual Case of Segmental Colitis in an Immunocompromised Host

**DOI:** 10.7759/cureus.1033

**Published:** 2017-02-16

**Authors:** Ilias P Papakonstantinou, Emmanuel A Andreadis

**Affiliations:** 1 4th Internal Medicine Department, “Evangelismos” General Hospital, Athens, Greece

**Keywords:** non-hodgkin lymphoma, double hit, colonic lymphoma, segmental colitis, gastrointestinal vasculitis, immunodeficiency, drug-induced lupus

## Abstract

In this report we describe a case of a 66-year-old woman who presented with right upper quadrant abdominal pain and bloody diarrhea. A workup revealed immunodeficiency, an immunologic profile with low complement levels resembling systemic lupus erythematosus, and a circumferential colonic wall lesion located in the ascending colon. After endoscopy and biopsy, the mass lesion was attributed to “double hit” diffuse large B-cell lymphoma, categorized as high grade large B-cell non-Hodgkin lymphoma according to the most recent revised 2016 World Health Organisation classification and considered to be a rare and highly aggressive tumor. The diagnosis of colonic lymphoma can be challenging due to a diversity of clinical presentation and requires a high index of suspicion. As the literature of such documented reports is limited, this case suggests further investigations.

Abbreviations: GI: gastrointestinal tract, DLBCL: diffuse large B cell lymphoma, DH: double hit lymphoma, SLE: systemic lupus erythematosus, ANA: antinuclear antibodies, anti-ssDNA: anti-single-stranded DNA, BCL: B-cell lymphoma protein, MUM-1/IRF4: multiple myeloma oncogene 1/interferon regulatory factor 4, HGBL: high grade B-cell lymphoma, anti-dsDNA: anti-double-stranded DNA.

## Introduction

Gastrointestinal (GI) lymphomas are uncommon malignancies [1-2]. Among the most frequent GI lymphoma subtypes, diffuse large B-cell lymphomas (DLBCLs) are the commonest, and around 5–10% of DLBCLs are ‘double hit’ lymphomas (DH) that represent rearrangements affecting MYC proto-oncogene and B-cell lymphoma protein 2 and/or 6 [2-4]. This is a unique case of DH GI lymphoma presenting with gastrointestinal symptoms and an immunologic profile resembling systemic lupus erythematosus (SLE), including positive antinuclear (ANA), anti-single-stranded DNA (anti-ssDNA), anti-histones antibodies and low complement levels. Informed consent was obtained from the patient for this study.

## Case presentation

A 66-year-old woman presented to the emergency department with a one-week history of abdominal pain, mainly located at the right upper quadrant. In the presenting days she reported bloody stools with no constitutional symptoms.

Her past medical history was significant for cryptogenic organising pneumonia and paroxysmal atrial fibrillation. Current medications included a tapering dose of methylprednisolone (at 8 mg/day), dabigatran, and flecainide. A physical examination revealed mild right upper quadrant and right flank tenderness with no rigidity, rebound, or guarding. The rest of the physical examination was unremarkable.

Laboratory investigations on admission showed normocytic normochromic anemia, leukopenia with lymphocytopenia, hypogammaglobulinaemia, as well as mild transaminitis and elevated lactic dehydrogenase. The blood cultures were negative.

Further workup and a computed tomography scan of the abdomen revealed significant circumferential thickening and stenosis of the colonic wall, located in the ascending colon proximal to the hepatic flexure extending in a continuous distribution further to the left colon. Enlarged lymph nodes were not found.

An endoscopic evaluation showed a near-obstructive, solid and edematous, fragmented-appearing mass lesion located at the splenic flexure and extending beyond. A biopsy of the mass was performed. A course of 5-aminosalicylic acid (mesalazine) for presumed inflammatory bowel disease was initiated.

The patient’s lymphocytopenia and hypogammaglobulinaemia indicated that the patient was immunocompromised. Further immunologic evaluation demonstrated significant T CD4+ and B CD19+ lymphocytopenia with no evidence of hematologic malignancy, significantly low levels of complement C4 and C3 indicative of complement activation, positive ANA: 1/160, anti-ssDNA and anti-histones antibodies (Table 1). The patient lacked any SLE clinical signs and could not meet the standard defined classification for diagnosis of SLE [5].

Meanwhile, a histopathological examination of the biopsy specimens was non-specific. The patient’s symptoms persisted and her clinical course deteriorated. The summary of the diagnostic results during workup was that the patient had undetermined segmental colitis, immunodeficiency, and an immunologic profile of SLE. The differential diagnosis included other rare conditions like the GI lymphoma [1] and mesenteric gastrointestinal vasculitis or drug-induced colitis, since both entities present with clinically apparent visceral involvement and/or bowel vasculitis [6].

From repeated gastrointestinal endoscopy and biopsy, infiltrative colonic B-cell lymphoma was determined. The lymphoma was categorized as unclassifiable DH type [3]. Immunohistochemical staining revealed the tumor to be positive for CD20, B-cell lymphoma protein 6 and 2 (BCL-6 and BCL-2), multiple myeloma oncogene 1/interferon regulatory factor 4 (MUM-1/IRF4) and negative for CD10, CD5. The Ki-67 proliferation rate was 90%. According to the most recently revised 2016 World Health Organisation classification, the tumors that were provisionaly characterized as DΗ lymphomas and harbor concurrent rearrangements of the MYC proto-oncogene and BCL2 and/or BCL6 are placed in a new definitive single category of rare and highly aggressive tumors, the high-grade B-cell lymphomas (HGBL) [7]. 

The patient was treated with the regimen R-CHOP (rituximab, cyclophosphamide, doxorubicin, vincristine, and prednisolone) and adjunctive intrathecal methotrexate for six cycles as she was evaluated unfit for more intensive, compared to R-CHOP, induction therapies [4]. After 18 months of the initial presentation, she is monitored on a regular basis as an outpatient and no further hospitalizations are required.

**Table 1:** Immune System Evaluation Serum immunoglobulins IgA and IgG were markedly reduced. Significantly low levels of complement C4 and C3 were found, indicative of complement activation. Antinuclear antibodies were 1/160 positive, while anti-double-stranded DNA antibodies and Anti-Sm antibodies were negative. Conversely, anti-ssDNA and anti-histones antibodies, were positive. Peripheral blood flow cytometry demonstrated significant T CD4+ and B CD19+ lymphocytopenia. CD4+ T, CD8+ T, and CD19+ B lymphocytes are in absolute values and percentage (%) of blood lymphocytes.

Test	Value	Normal limits
*Complement *		
C3	53.7	63-158 (mg/dL)
C4	6.5	14-33 (mg/dL)
*Immunoglobulins *		
IgG	172	690-1618 (mg/dL)
IgA	23.8	72-400 (mg/dL)
IgM	323	40-235 (mg/dL)
IgE	85	10-100 (IU/mL)
*Antibodies *		
RF	<10.2	<20 (IU/mL)
ANA	1:160(+)	<1:160
Anti-centromere	(-)	
Anti- dsDNA	(-)	
Anti-Sm	(-)	
Anti-ssDNA	28.4	<15
Anti-Histones	2.9	<2
*Immunophenotype*		
CD4+ T	242 /51%	663–1477 cells/μL
CD8+ T	11 /2.36%	342–754 cells/μL
CD19 B	40 /8.3%	150 - 400 cells/μL

## Discussion

This case highlights the need for increased awareness in patients presenting with segmental colitis and immunodeficiency when remission of the disease has not been achieved with the treatment of colitis. The deterioration of our patient's clinical condition and the laboratory findings strongly pointed to an underlying disease that was not obvious at the initial presentation.

In order to differentiate drug-induced lupus that shares several clinical and serologic features with SLE, the antiarrythmic drug flecainide was discontinued, but the serologic features of lupus did not resolve. It is known that in drug-induced lupus, antibodies to histones and anti-ssDNA are common but are also seen in SLE and thus do not distinguish from SLE [8]. Furthermore, complement levels are usually normal in drug-induced lupus rather than low as were in our case.

Of interest, autoimmune and chronic inflammatory conditions, predispose to immune dysregulation and imbalance of normal B-cell activation leading eventually to an increased occurrence of lymphoma [9]. Among SLE patients in particular, increased expression of cytokine pathways, the proliferation-inducing ligand (APRIL) and B-cell activating factor (BAFF) may explain the pathogenic B-cell activation and offer a common link between autoimmunity and lymphoma [10].

Finally, the most relevant clinical features of colonic lymphomas include age >50 years, high frequency of chronic immunosuppression, location proximal to the hepatic flexure, and long-segment circumferential extension [11-12]. Early detection and therapy of colonic lymphoma has a significant impact in the disease progression, and it is a major determinant leading, therefore, to a better prognosis [13].

## Conclusions

In conclusion, as evidence relating DH lymphoma with SLE is lacking, the coexistence of these two entities makes this presentation very unusual. Even though this case may suggest an autoimmune etiology contributing to the pathogenesis of DH lymphoma, it is too limited to be conclusive. Further studies are needed to elucidate the role of autoimmunity in the pathogenesis of DH lymphoma.
